# Rapid Identification of QTL for Mesocotyl Length in Rice Through Combining QTL-seq and Genome-Wide Association Analysis

**DOI:** 10.3389/fgene.2021.713446

**Published:** 2021-07-19

**Authors:** Yamei Wang, Jindong Liu, Yun Meng, Hongyan Liu, Chang Liu, Guoyou Ye

**Affiliations:** ^1^Shenzhen Branch, Guangdong Laboratory for Lingnan Modern Agriculture, Genome Analysis Laboratory of the Ministry of Agriculture and Rural Affairs, Agricultural Genomics Institute at Shenzhen, Chinese Academy of Agricultural Sciences, Shenzhen, China; ^2^CAAS-IRRI Joint Laboratory for Genomics-Assisted Germplasm Enhancement, Agricultural Genomics Institute at Shenzhen, Chinese Academy of Agricultural Sciences, Shenzhen, China; ^3^College of Tropical Crops, Hainan University, Haikou, China; ^4^Rice Breeding Innovation Platform, International Rice Research Institute, Metro Manila, Philippines

**Keywords:** candidate gene, GWAS, mesocotyl, QTL-seq, rice

## Abstract

Mesocotyl is a crucial organ for pushing buds out of soil, which plays a vital role in seedling emergence and establishment in direct-seeded rice. Thus, the identification of quantitative trait loci (QTL) associated with mesocotyl length (ML) could accelerate genetic improvement of rice for direct seeding cultivation. In this study, QTL sequencing (QTL-seq) applied to 12 F_2_ populations identified 14 QTL for ML, which were distributed on chromosomes 1, 3, 4, 5, 6, 7, and 9 based on the Δ(SNP-index) or *G*-value statistics. Besides, a genome-wide association study (GWAS) using two diverse panels identified five unique QTL on chromosomes 1, 8, 9, and 12 (2), respectively, explaining 5.3–14.6% of the phenotypic variations. Among these QTL, seven were in the regions harboring known genes or QTLs, whereas the other 10 were potentially novel. Six of the QTL were stable across two or more populations. Eight high-confidence candidate genes related to ML were identified for the stable loci based on annotation and expression analyses. Association analysis revealed that two PCR gel-based markers for the loci co-located by QTL-seq and GWAS, *Indel-Chr1:18932318* and *Indel-Chr7:15404166* for loci *qML1.3* and *qML7.2* respectively, were significantly associated with ML in a collection of 140 accessions and could be used as breeder-friendly markers in further breeding.

## Introduction

Rice (*Oryza sativa*) is one of the most important food crops in the world, providing more than 21% of the food for the world’s population. Maintaining a higher and stable yield is of great importance for food security, especially in developing countries in Asia^1^. Transplanting and direct seeding are two major rice planting patterns. Direct seeding refers to the process of establishing seedlings into puddled or submerged soil without the transplanting process (Kumar and Ladha, 2011; Zhan et al., 2020). Compared with traditional rice transplanting, direct seeding is water-efficient and labor-saving (Kato and Katsura, 2014; Liu et al., 2015; Ohno et al., 2018). However, direct seeding is also facing problems such as low seedling emergence rate, poor seedling establishment, weed infestation, and high crop lodging rate (Mahender et al., 2015; Lee et al., 2017). Mesocotyl, an organ between the coleoptile node and the basal part of the seminal root in rice seedlings, plays a key role in pushing buds out of deep water or soil for successful seedling establishment during germination (Zhan et al., 2020). Thus, varieties with longer mesocotyl can be used to partially overcome the problems faced by direct seeding (Lee et al., 2017; Zhan et al., 2020).

Mesocotyl length (ML) is a quantitative trait controlled by multiple minor effect genes (Li et al., 2017; Sun et al., 2018; Liu et al., 2020; Zhan et al., 2020). Up to now, over 40 quantitative trait loci (QTL) on 12 rice chromosomes have been identified, which could explain 5.7–27.8% of the phenotypic variation (Cao et al., 2002; Liu et al., 2020; Rohilla et al., 2020; Zhan et al., 2020). Four genes were cloned: *OsGY1* (Xiong et al., 2017), *OsGSK2* (Sun et al., 2018), *OsSMAX1* (Zheng et al., 2020), and *OsPAO5* (Lv et al., 2021). Recently, genome-wide association study (GWAS) based on linkage disequilibrium (LD) has been widely applied to identify marker–trait associations (MTAs) for complex agronomic traits (Meng et al., 2016; Zhan et al., 2020; Liu et al., 2021). Compared with traditional biparental linkage mapping, GWAS provides a more representative gene pool because all the historical meiotic events can be counted from a diverse panel and is an efficient tool that bypasses the time and expand to the developing population (Flint-Garcia et al., 2003; Breseghello and Sorrells, 2006; Zhu et al., 2008). Now, GWAS has been adopted to investigate a range of complex traits in crops, including disease resistance (Liu et al., 2017; Resende et al., 2017; Prodhomme et al., 2020), grain quality (Yang et al., 2020), yield-related traits (Meng et al., 2016), salt tolerance (Zhang et al., 2020; Ponce et al., 2021), and microelements (Chen et al., 2019; Liu et al., 2020).

To rapidly identify QTL in plants, QTL sequencing (QTL-seq), an effective and economic approach combining the traditional bulk segregant analysis (BSA) and high-throughput whole-genome resequencing, has been developed (Takagi et al., 2013). For QTL-seq, a mapping population was firstly generated by crossing two cultivars showing the extreme target phenotypes, and then two DNA pools from individuals with extreme phenotype in the population and two pools from the parents were constructed and sequenced (Takagi et al., 2013). This method has been successfully used to rapidly identify QTL for a number of traits in rice, such as blast disease resistance and seedling vigor (Takagi et al., 2013), cold tolerance (Luo et al., 2018), cooked grain elongation (Arikit et al., 2019), and low phosphorus tolerance (Nishida et al., 2018).

In this study, QTL-seq (applied to 12 F_2_ populations) and GWAS (applied to three diverse panels) were used to rapidly identify QTL for ML in rice. Candidate genes for the important QTL were investigated and two breeder-friendly molecular markers were developed for the loci co-located by GWAS and QTL-seq.

## Materials and Methods

### Plant Materials

**FIGURE 1:**
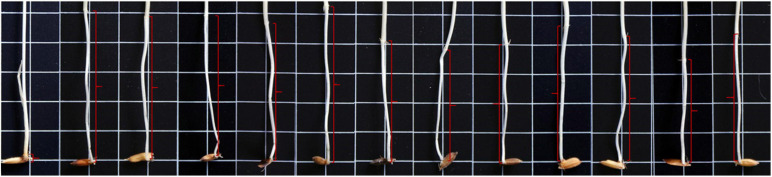
Mesocotyl (*red brackets*) length of “IR 145” and the 12 male parents of the F_2_ populations. The 13 individuals were “IR 145” (0.18 cm) and the corresponding male parents of Pop1 to Pop12 (from *left* to *right*) were as follows: 79 (5.13 cm), Bamla Suffaid 32 (4.80 cm), M 136–20 (4.67 cm), Cash (4.75 cm), Balam 2 (4.83 cm), Kalasu (4.20 cm), Black 28–573 (3.39 cm), Bhahuri (4.18 cm), Changai (4.74 cm), Basmati 385 (4.68 cm), IR 64 (3.48 cm), and BR 11 (4.24 cm).

The rice variety “IR 145” with short mesocotyl (0.18 cm) was crossed with 12 accessions with long mesocotyl (ranging from 3.39 to 5.13 cm) (Table 1 and Figure 1) to develop the F_2_ populations for QTL-seq. These populations were named as Pop1 to Pop12, respectively. Two diverse panels, XI-1A (147 accessions) and AUS (171 accessions) of the 3K Resequencing Project (Wang et al., 2018), were used for GWAS (Supplementary Tables 1, 2). Most of the accessions in XI-1A originated from China (Supplementary Table 1), whereas accessions in the AUS group mainly came from Bangladesh, India, and Pakistan (Supplementary Table 2). One diverse panel consisting of 140 accessions originated from the XI-1B of 3K Resequencing Project (Supplementary Table 3), mainly from China, which were used to validate the effectiveness of the markers developed based on QTL-seq and GWAS (Wang et al., 2018).

**TABLE 1:** Details of the male parents of the 12 F_2_ populations.

Population name	Male parent name	Mesocotyl length (cm)	Origin
Pop1	79	5.13	Pakistan (XI-1A)
Pop2	Bamla Suffaid 32	4.80	– (XI-1A)
Pop3	M 136-20	4.67	India (XI-1A)
Pop4	Cash	4.75	Bangladesh (AUS)
Pop5	Balam 2	4.83	Bangladesh (AUS)
Pop6	Kalasu	4.20	Philippines (XI-1A)
Pop7	Black 28-573	3.39	Bangladesh (AUS)
Pop8	Bhahuri	4.18	Bangladesh (AUS)
Pop9	Changai	4.74	Bangladesh (AUS)
Pop10	Basmati 385	4.68	India (XI-1A)
Pop11	IR64	3.48	Philippines (AUS)
Pop12	BR11	4.24	Bangladesh (AUS)

### Phenotyping of ML

To evaluate ML, high-quality seeds were sown in a plastic tray containing 6-cm-deep soil according to Liu et al. (2020). The plastic tray has the following specifications: 50 holes, each with a size of 9.5 cm in depth, 4.5 cm in top diameter, and 2.1 cm in bottom diameter. After sowing, the seeds were covered with nutrient soil until the hole was filled up. Then, the tray was placed in a plastic pallet with 3-cm-deep nutrient soil and the system was kept in a dark incubator (30°C/65% relative humidity). The soil in each pallet was kept water saturated for seed germination and seedling growth. Three days after all of the seeds germinated (about 7–10 days after sowing), the seedlings from each hole were carefully excavated and rinsed with double-distilled water (ddH_2_O) for ML measurement with ImageJ software^2^. For QTL-seq, a total of 720 individuals of each population were sown. Meanwhile, two parents of the corresponding population were also planted for ML measurement and sampling. For GWAS, 15 seeds from each accession were used to evaluate the ML, and mean of two replications was used for statistical analysis.

### Genotyping

For QTL-seq, the genomic DNA from seedling leaf was isolated according to the modified cetyl trimethylammonium bromide (CTAB) method (Doyle and Doyle, 1987). For a specific F_2_ population, DNA samples with equal amounts from 50 individuals with extremely long mesocotyl were selected to generate the long mesocotyl (LM) bulk, whereas those from 50 individuals with extremely short mesocotyl were used to form the short mesocotyl (SM) bulk. The “IR 145” library and 12 libraries from the male parents, as well as 24 extreme libraries, were sequenced using the Illumina HiSeq 2500 platform (Illumina, Inc., San Diego, CA, United States) by Berry Genomics Corporation, Beijing, China^3^. The paired-end read data (PE150) with a sequencing depth of approximately 50× of the rice genome (∼400 Mb) were generated. The clean reads were obtained by removing reads with adaptors, with a ratio of *N* larger than 10%, and those of low quality (the number of bases with *Q* ≤ 3 accounts for more than 50% of the whole reads).

The clean reads were aligned to Nipponbare RefSeq (IRGSP-1.0)^4^ using BWA-MEM (release 0.7.10) (Li and Durbin, 2009). Then, the mapped reads were sorted and the duplicate ones were removed by Picard tools^5^. The variants for each accession were called for single nucleotide polymorphism (SNP) detection and annotation by GATK Best Practices (release 3.2-2;^6^). For the GWAS panel, the genotype data for XI-1A, AUS, and XI-1B were obtained from 3K Resequencing Project with Nipponbare IRGSP-1.0 as the reference genome and at about 11.5× coverage with an average mapping coverage of 92% (Wang et al., 2018).

### QTL-seq Analysis

Reads from the LM bulk and SM bulk of an F_2_ population were aligned to the “IR 145” variants, respectively. The SNP-index was calculated at each SNP site according to Takagi et al. (2013). The polymorphic sites were then filtered according to the following criteria: (1) the SNP-index values in both bulks were < 0.2 and the SNP sequencing depths were < 7; (2) the SNP-index in either bulk was missing; and (3) GQ < 20 in either bulk. For both bulks, the average SNP-index in each chromosome was presented using a sliding window approach with a 200-kb window size to generate the SNP-index plots for all chromosomes. The Δ(SNP-index) was then calculated by subtracting the SNP-index of LM bulk with that of the SM bulk. The regions in which the average Δ(SNP-index) at a locus was significantly greater than the surrounding regions at 95% confidence interval were considered as candidate regions (Takagi et al., 2013).

### Population Structure and LD Decay Analysis

The population structures for the AUS and XI-1A panels were analyzed using 10,000 polymorphic SNP markers with Admixture 1.3.0 (Alexander et al., 2009). Five independent runs for each *K* value from 2 to 7 were performed based on an admixture model. An *ad hoc* quantity statistic, Δ*K*, based on the rate of change in log probability of data between successive *K* values was used to predict the real number of subpopulations. Principal component analysis (PCA) and neighbor-joining (NJ) trees for AUS, XI-1A, and XI-1B were also used to validate population stratification with Tassel v5.0 (Bradbury et al., 2007). The LD among markers was calculated using the full matrix and sliding window options in Tassel v5.0 with 10,000 evenly distributed SNP markers. The squared allele frequency correlation, *r*^2^, values were plotted against physical distance and a LOESS (locally weighted smoothing) curve was fitted to the plot to show the association between LD decay and physical map distance. The intersection of the fitted curve of *r*^2^ values with threshold of 95th percentile in the distribution of *r*^2^ was considered as the estimate of the LD range (Breseghello and Sorrells, 2006).

### Genome-Wide Association Analysis

Previous studies have reported the details of the genotype data, LD decay, PCA, and the population structure for the XI-1A and AUS panels (Wang et al., 2018). Associations between the genotypic and phenotypic data were analyzed using the kinship matrix in an MLM (mixed linear model) by GAPIT^7^ based on R 3.6.1 (Lipka et al., 2012) to control background variations and eliminate spurious MTAs. Since the Bonferroni–Holm correction for multiple testing (*α* = 0.05) was too conserved, markers with an adjusted −log_10_(*P*-value) ≥ 4.0 were regarded as the significant ones.

### Candidate Gene Identification and Gene Expression Analysis

Candidate genes for the loci consistently identified in two or more populations were identified. The following steps were conducted to identify the candidate genes for important QTL. Firstly, excavate all the genes located in the LD block region around the peak SNP (±150 kb based on previous LD decay analysis) of each important QTL from the MSU Rice Genome Annotation Project^8^. Then, all available SNPs located inside of these genes were searched. The genes (except for the expressed protein, hypothetical protein, transposon protein, and retrotransposon protein) with SNPs in the coding region that could further lead to sense mutations were considered as candidate genes. A candidate gene with identical SNPs or InDels in the male parent of the co-localized population was further selected. As mesocotyl elongation is highly regulated by various phytohormones, including strigolactones (SLs), cytokinins (CTKs), brassinosteroids (BRs), abscisic acid (ABA), jasmonates (JAs), gibberellins (GAs), and auxins (IAAs) (Watanabe and Takahashi, 1999; Cao et al., 2005; Hu et al., 2014; Xiong et al., 2017; Sun et al., 2018; Zheng et al., 2020; Lv et al., 2021), those genes involved in phytohormone metabolism were regarded as high-confidence candidate genes for mesocotyl elongation.

Quantitative real-time PCR (qRT-PCR) was conducted to test expression differences of the candidate genes in parents of the corresponding F_2_ population. The mesocotyl section of “IR 145” and the male parent were sampled for RNA extraction at 52 h after germination before the coleoptile was unearthed. Total RNA was extracted according to the Trizol method. Complementary DNA (cDNA) was synthesized with the HiScript II 1st Strand cDNA Synthesis Kit (Vazyme, Nanjing, China) and then diluted 5–10 times with sterile double distilled water. The primers were designed with Primer Premier 5.0 software^9^. PCR procedure was conducted in a volume of 20 μl, containing 2 μl cDNA, 0.4 μl of each primer (in micromolars), and 10 μl ChamQ Universal SYBR qPCR Master Mix. The reaction was conducted in the ABI StepOnePlus Real-Time PCR System with Tower (ABI, Waltham, MA, United States). The gene expression level was analyzed with 2^–ΔΔCT^ method. *OsActin1* was used as internal control to normalize the expression levels of different samples. All assays were performed in two independent experiments, each with three repetitions.

### Development of Allele-Specific Markers

For InDel markers, the primers were designed by Primer Premier 5 software (Lalitha, 2000), with a pair of primers spanning the InDel region and the amplified fragment size was set to no more than 10 times that of the InDel. A PCR procedure was implemented for the two markers. The PCR procedure was conducted in a volume of 25.0 μl, which includes 12.5 μl of 2× Taq Master Mix (Vazyme, Nanjing, China), 2 μl of template DNA, 1 μl of each primer (10 μM), and 8.5 μl of ddH_2_O. The PCR program was set as follows: an initial denaturation at 94°C for 5 min, 35 cycles of denaturation at 94°C for 30 s, annealing at 55°C for 30 s, extension at 72°C for 30 s, and a final extension at 72°C for 10 min.

### Statistical Analysis

One-way analysis of variance (ANOVA) was performed using SPSS Statistics 17.0^10^. Tukey’s multiple test was employed for multiple comparisons (^∗^*P* < 0.05 and ^∗∗^*P* < 0.01).

## Results

### ML Exhibited Continuous Variation in the F_2_ Populations and GWAS Panels

ML of the common parental line “IR 145” was 0.18 cm, while the other 12 parental lines had MLs ranging from 3.39 to 5.13 cm (Table 1 and Figure 1). The average MLs of 12 F_2_ populations were: 0.85 (Pop1, 0–5.03 cm), 0.75 (Pop2, 0–4.61 cm), 0.78 (Pop3, 0–4.98 cm), 0.91 (Pop4, 0–5.71 cm), 0.72 (Pop5, 0–4.31 cm), 0.69 (Pop6, 0–5.32 cm), 0.78 (Pop7, 0–4.98 cm), 0.83 (Pop8, 0–5.25 cm), 0.81 (Pop9, 0–4.91 cm), 0.70 (Pop10, 0–5.22 cm), 0.95 (Pop11, 0.2–5.13 cm), and 0.84 (Pop12, 0–5.93 cm) (Supplementary Figure 1). The MLs ranged from 0.0 to 3.41 cm, with an average of 0.85 cm in the XI-1A panel (Supplementary Table 1 and Supplementary Figure 2), whereas it ranged from 0.2 to 4.42 cm with an average of 2.41 cm in the AUS panel (Supplementary Table 2 and Supplementary Figure 2). Continuous variation with transgressive segregation on both sides was observed across both populations with approximately normal distributions (Supplementary Figures 1, 2).

### QTL Identified by QTL-seq

In total, 85–112 Gb clean reads were obtained, and all the Q30 reached 85%. The average sequencing depth was 46.83×, and the average mapped ratio and genome coverage have reached 96.5 and 95.4%, respectively (Supplementary Table 3). A total of 5,128,693–6,984,215 SNPs were detected in 12 F_2_ populations (Supplementary Table 4). The marker density ranged from 14.2 markers/kb (chromosome 3 of Pop11) to 22.8 markers/kb (chromosome 7 of Pop1), with an average of 18.2 markers/kb.

**FIGURE 2:**
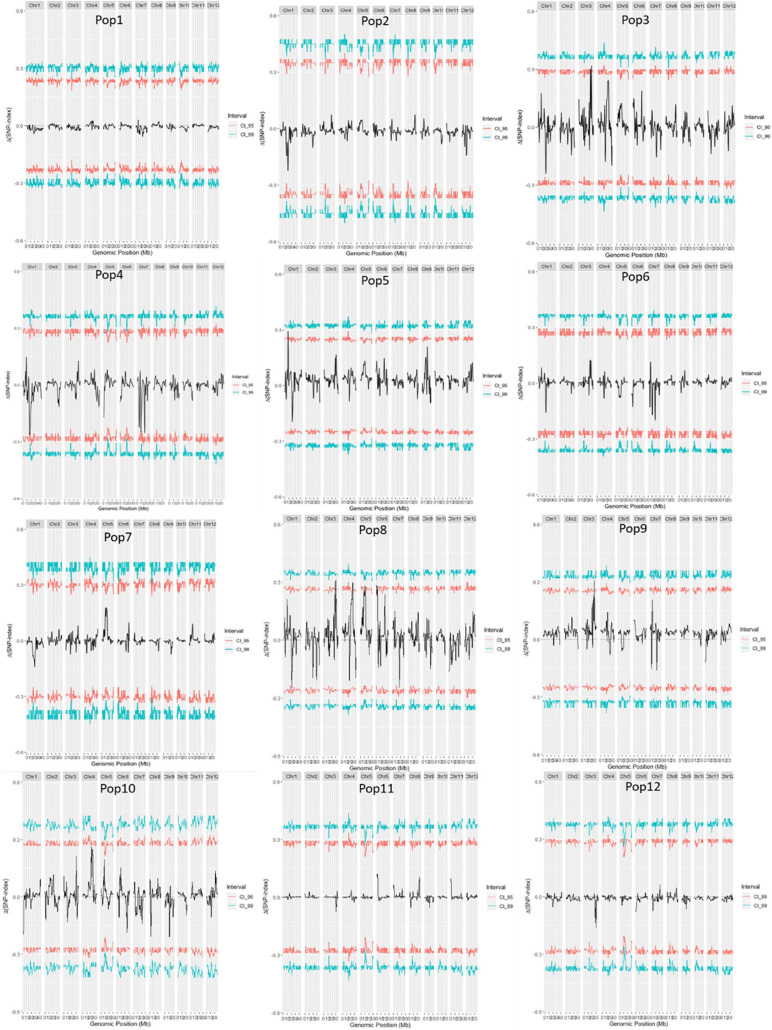
Δ(SNP-index) of 12 F_2_ populations. The female parents of the 12 F_2_ populations were: 79 (Pop1), Bamla Suffaid 32 (Pop2), M 136–20 (Pop3), Cash (Pop4), Balam 2 (Pop5), Kalasu (Pop6), Black 28–573 (Pop7), Bhahuri Kalasu (Pop8), Changai Kalasu (Pop9), Basmati 385 Kalasu (Pop10), IR 64 Kalasu (Pop11), and BR 11 Kalasu (Pop12).

**FIGURE 3:**
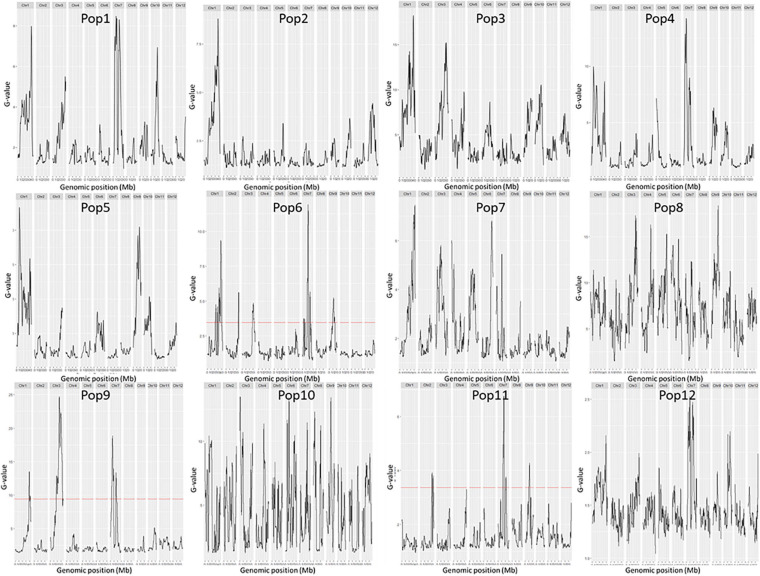
*G*-values of the 12 F_2_ populations. The female parents of the 12 F_2_ populations were: 79 (Pop1), Bamla Suffaid 32 (Pop2), M 136–20 (Pop3), Cash (Pop4), Balam 2 (Pop5), Kalasu (Pop6), Black 28–573 (Pop7), Bhahuri Kalasu (Pop8), Changai Kalasu (Pop9), Basmati 385 Kalasu (Pop10), IR 64 Kalasu (Pop11), and BR 11 Kalasu (Pop12).

A total of 14 regions were identified by the Δ(SNP-index) value or the *G*-value (Table 2 and Figures 2, 3). These loci were located on chromosomes 1, 3–7, and 9, and the interval size ranged from 0.43 to 3.54 Mb (Table 2). Four unique QTL for ML—*qML1.1* (Pop5, 6.57–8.12 Mb), *qML1.2* (Pop4, 14.21–17.32 Mb), *qML1.3* (Pop2, Pop3, Pop6, and Pop8, 18.59–21.12 Mb), and *qML1.4* (Pop4 and Pop 12, 36.59–39.67 Mb)—were identified on chromosome 1. Three adjacent QTL were isolated on chromosome 3. Of these, *qML3.1* was detected in Pop3 and located at the interval of 25.12–27.50 Mb, whereas *qML3.2* was discovered in Pop8 and Pop12 and fixed at 27.21–32.30 Mb. *qML3.3*, unearthed in Pop9, was set at the interval of 35.72–36.15 Mb. Two loci were found to exist on chromosome 4. Of these, *qML4.1* was recognized at Pop10 and situated at the interval of 19.59–21.92 Mb, whereas *qML4.2*, fixed at 25.50–27.13 Mb, was discovered at Pop8. On chromosome 5, only *qML5.1* was identified from Pop8 and located at the 8.99- to 11.03-Mb region. Another genomic region (2.59–5.12 Mb) identified on chromosome 6 with Δ(SNP-index) plots greater than the statistical confidence intervals (*P* < 0.05) was named as *qML6.1*. Two adjacent QTL (*qML7.1* and *qML1.2*) were identified on chromosome 7. Besides, *qML7.1* (4.35–8.65 Mb) was identified in Pop4, Pop6, and Pop9, whereas *qML7.2* (13.69–18.49 Mb) was identified in Pop4, Pop6, Pop8, Pop9, Pop11, and Pop12. In addition, only one locus, *qML9.1* (11.53–15.28 Mb), was identified on chromosome 9 in Pop5 and Pop12.

**TABLE 2:** QTL-seq for mesocotyl length in 12 F_2_ populations.

Name	Chromosome	Start (Mb)	End (Mb)	Length (Mb)	Position of max. *G*-value (Mb)	Position of max. Δ(SNP-index) (Mb)	Population	References
*qML1.1*	1	6.57	8.12	1.55	7.21	7.32	5	–
*qML1.2*	1	14.21	17.32	3.11	14.22	14.51	4	Lu et al., 2016
*qML1.3*	1	18.59	20.15	1.56	–	19.93	3	Ouyang et al., 2005
	1	18.51	20.36	1.85	20.01	20.12	8	
	1	19.88	21.12	1.24	–	19.83	6	
	1	20.04	21.12	1.08	20.34	20.34	2	
*qML1.4*	1	36.59	39.01	2.42	37.92	38.57	12	Xiong et al., 2017
	1	38.32	39.67	1.35	39.26	38.39	4	
	1	37.58	39.30	1.82	38.05	38.05	9	
*qML3.1*	3	25.12	27.5	2.38	25.24	26.53	3	–
*qML3.2*	3	27.21	30.13	2.92	29.50	29.84	8	Zhao et al., 2018
	3	29.51	32.3	2.79	31.32	31.36	12	
*qML3.3*	3	35.72	36.15	0.43	35.86	36.04	9	–
*qML4.1*	4	19.59	21.92	2.33	21.37	21.25	10	–
*qML4.2*	4	25.50	27.13	1.63	25.58	25.92	8	–
*qML5.1*	5	8.99	11.03	2.04	10.16	10.21	8	–
*qML6.1*	6	2.59	5.12	2.53	4.99	3.87	8	–
*qML7.1*	7	4.98	8.52	3.54	7.90	8.84	4	–
	7	4.35	7.56	3.21	5.63	6.03	9	–
	7	5.52	8.65	3.13	7.31	7.92	6	–
*qML7.2*	7	13.69	16.25	2.56	15.82	15.88	11	Ouyang et al., 2005; Zhao et al., 2018; Liu et al., 2020
	7	14.99	17.12	2.13	15.85	17.13	12	
	7	14.89	16.52	1.63	16.37	16.12	8	
	7	15.45	16.98	1.53	15.84	15.03	9	
	7	15.58	18.24	2.66	17.23	16.92	6	
	7	16.37	18.49	2.12	17.68	17.21	4	
*qML9.1*	9	11.53	13.54	2.01	12.29	12.25	12	–
	9	11.99	15.28	3.29	15.13	13.56	5	–

### QTL Identified by GWAS

In total, 2,338,386 SNPs were left and employed for GWAS. The chromosome size varied from 22.8 Mb for chromosome 9 to 43.2 Mb for chromosome 1. These markers spanned a physical distance of 373 Mb, with an average density of 6.25 markers/kb.

**FIGURE 4:**
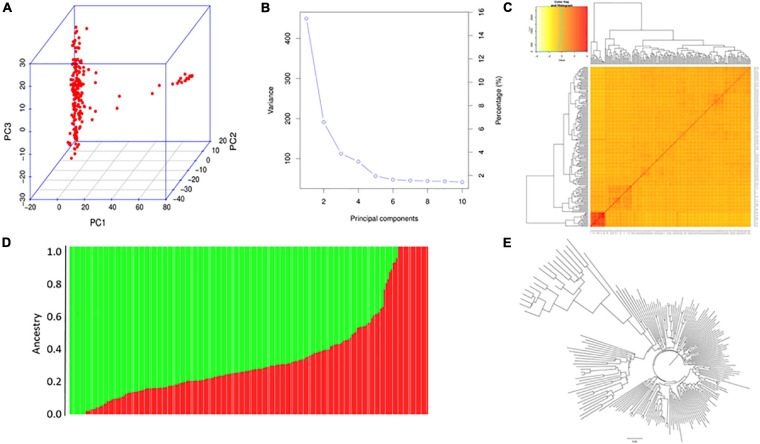
Population analysis for the XI-1A panel. **(A)** Principal component analysis (PCA) plots. **(B)** Total variation explained by the principal components (PCs). **(C)** Kinship relatedness. **(D)** Two subgroups inferred by structure analysis. **(E)** Neighbor-joining (NJ) tree.

PCA of XI-1A panel indicated that the top three principal components (PCs) could explain 15.8, 6.2, and 4.1% of total variation (Figures 4A,B), respectively, and this panel consists of two subgroups (Figure 4C). The NJ tree showed that the two clades represented two subpopulations (Figure 4E). Structure analysis indicated that XI-1A could be divided into two subgroups, subgroup 1 and subgroup 2 (Figure 4D), whose characterizations were largely consistent with their geographic origins. The XI-1A-1 accessions were mainly from South China, whereas those of XI-1A-2 were mainly from the Yangtze River plain, China. Also, admixture accessions were observed in the present study (Figure 4).

**FIGURE 5:**
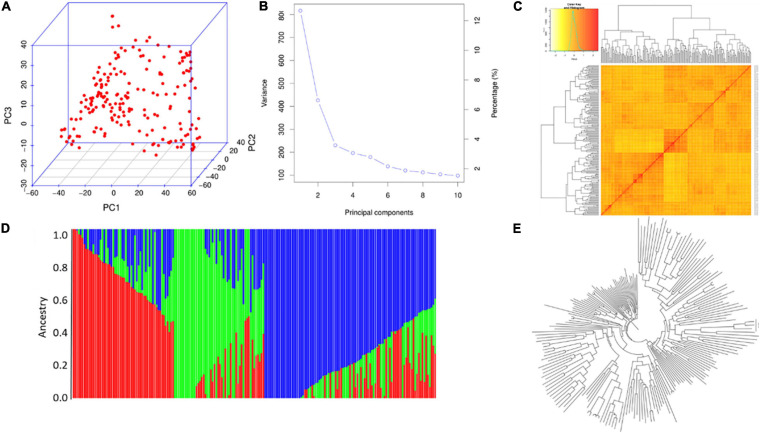
Population analysis for the AUS panel. **(A)** Principal component analysis (PCA) plots. **(B)** Total variation explained by the principal components (PCs). **(C)** Kinship relatedness. **(D)** Three subgroups inferred by structure analysis. **(E)** Neighbor-joining (NJ) tree.

For the AUS panel, PCA indicated that the top three PCs could explain 13.5, 6.5, and 4.2% of the total variation, respectively (Figure 5A,B). Structure analysis indicated that the AUS panel could be divided into three subgroups, AUS-1, AUS-2, and AUS-3 (Figure 5C,D), whose characterizations were largely consistent with their geographic origins and the results of PCA (Figure 5A) and NJ tree analysis (Figure 5E), which showed three clades in this panel. AUS-1, AUS-2, and AUS-3 mainly include accessions from Bangladesh, India, and Pakistan, respectively. The LD decay along the physical distances for the XI-1A and AUS panels are shown in Supplementary Figure 3; the corresponding LD decay distance was about 150 and 175 kb, respectively.

**FIGURE 6:**
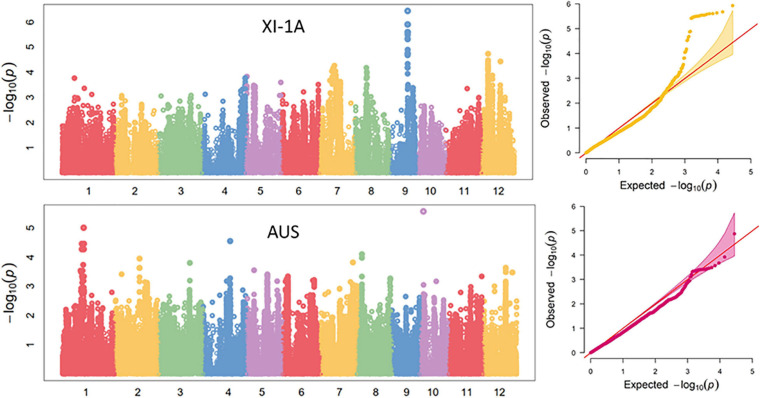
Genome-wide association study (GWAS) for mesocotyl length in the XI-1A and AUS panels.

Manhattan plots for the markers significantly associated with ML are shown in Figure 6. For the XI-1A panel, three unique loci located on chromosomes 8 (*qML-XI-1A-8.1*, 8.98–9.38 Mb), 9 (*qML-XI-1A-9.1*, 14.52–14.58 Mb), and 12 (*qML-XI-1A-12.1*, 5.58–5.76 Mb) were detected, which explained 5.5–11.3% of the ML variance (Table 3 and Supplementary Table 5). Notably, *qML-XI-1A-9.1* overlapped with the *qML9.1* (11.53–15.28 Mb) identified in Pop5 and Pop12 (Table 2). In the AUS panel, two unique loci located on chromosomes 1 (*qML-AUS-1.1*, 16.03–17.82 Mb) and 12 (*qML-AUS-12.1*, 18.29–18.50 Mb) were detected, which explained ML variations of 5.3 and 14.6%, respectively (Table 3 and Supplementary Table 6). Among which, the *qML-AUS-1.1* locus overlapped with the *qML1.2* (14.21–17.32 Mb) identified in Pop4 (Table 2 and Table 3).

**TABLE 3:** GWAS for mesocotyl length in the XI-1A and AUS panels.

Population	Locus name	Chromosome	Peak	Interval (Mb)	Peak	*R*^2^ (%)	Others
			Position (bp)		*P*-value		
XI-1A	*qML-XI-1A-8.1*	8	8,982,811	8.98–9.38	2.81E-05	6.69	
	*qML-XI-1A-9.1*	9	14,572,632	14.52–14.58	7.39E-07	11.27	*qML9.1*
	*qML-XI-1A-12.1*	12	5,716,291	5.58–5.76	0.000025	6.75	
AUS	*qML-AUS-1.1*	1	17,551,160	16.03–17.82	9.82E-06	14.57	Lu et al., 2016; *qML1.2*
	*qML-AUS-12.1*	12	18,365,206	18.29–18.50	2.28E-04	6.52	Rohilla et al., 2020

### Candidate Genes in the Important QTL Regions

The genes located in the LD block region around the peak SNP (±150 kb based on previous LD decay analysis) of each important QTL were excavated from the MSU Rice Genome Annotation Project^8^. Then, all available SNPs located inside of these genes were searched. Fifty-seven genes (except for the expressed protein, hypothetical protein, transposon protein, and retrotransposon protein) with SNPs in the coding region that lead to sense mutations were considered as candidate genes (Supplementary Table 7). A candidate gene with identical SNPs or InDels in the male parents of the 12 F_2_ populations showing overlapping QTL was further selected. In total, 12 genes were screened by this method.

**FIGURE 7:**
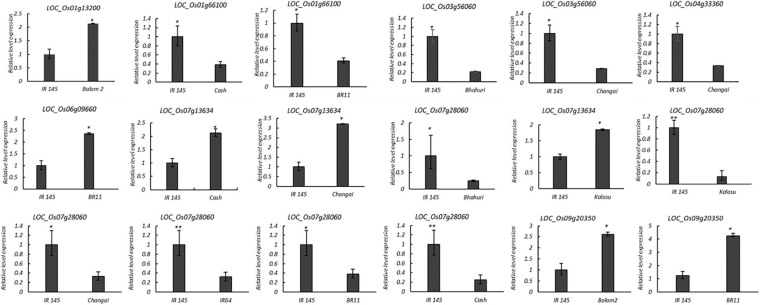
Expression difference of the candidate genes for mesocotyl elongation by qRT-PCR. The *X*-axis represents the two parents of the F_2_ population, whereas the *Y*-axis represents the relative expression of each gene to *β-actin* in mesocotyl tissue. Relative transcription levels were calculated by the 2^– ΔΔCT^ method. Significance at ^∗^*P* ≤ 0.05 and ^∗∗^*P* ≤ 0.01.

The expressions of 12 candidate genes in the parents of the corresponding F_2_ populations were detected using qRT-PCR (Figure 7 and Supplementary Table 8). Four genes, *LOC_Os01g36580*, *LOC_Os01g67670*, *LOC_Os04g44240*, and *LOC_Os07g08540*, showed no significant differences between the extreme ML parental accessions. Four genes—*LOC_Os01g13200*, *LOC_Os06g09660*, *LOC_Os07g13634*, and *LOC_Os09g20350*—showed more than 2.0- to 4.9-fold higher expressions in the long mesocotyl accessions compared to the short ones. Four genes, namely, *LOC_Os01g66100*, *LOC_Os03g56060*, *LOC_Os04g33360*, and *LOC_Os07g28060*, showed more than 1.9- to 7.6-fold lower expressions in the long mesocotyl accessions (Figure 7).

The eight genes showing obvious differences involved in the biological metabolism of phytohormones, cell elongation, and division were selected as the high-confidence candidate genes for mesocotyl elongation (Table 4). *LOC_Os01g13200* for *qML1.1* related to abscisic acid insensitive 8, *LOC_Os01g66100* from *qML1.4* encodes gibberellin oxidase, *LOC_Os03g56060* for *qML3.2* is a member of the cellulose synthase-like family, *LOC_Os04g33360* for *qML4.1* encodes gibberellin 2-beta-dioxygenase, *LOC_Os06g09660* for *qML6.1* is an auxin response factor, *LOC_Os07g13634* for *qML7.1* (7,815,442 bp) encodes cytokinin-*N*-glucosyltransferase 1, *LOC_Os07g28060* for *qML7.2* is an ethylene receptor, and *LOC_Os09g20350* for *qML9.2* is an ethylene-responsive transcription factor.

**TABLE 4:** Candidate genes for mesocotyl length by QTL-seq and GWAS.

Loci	Candidate gene	Start (bp)	End (bp)	Functional annotation
*qML1.1*	*LOC_Os01g13200*	7,352,175	7,357,620	Abscisic acid insensitive 8
*qML1.4*	*LOC_Os01g66100*	38,382,382	38,385,504	Gibberellin 20 oxidase 2
*qML3.2*	*LOC_Os03g56060*	31,930,541	31,934,624	CSLC9—cellulose synthase-like family C
*qML4.1*	*LOC_Os04g33360*	20,200,072	20,201,885	Gibberellin 2-beta-dioxygenase 7
*qML6.1*	*LOC_Os06g09660*	4,926,492	4,932,177	Auxin response factor
*qML7.1*	*LOC_Os07g13634*	7,815,442	7,832,311	Cytokinin-*N*-glucosyltransferase 1
*qML7.2*	*LOC_Os07g28060*	16,368,462	16,368,840	Ethylene receptor
*qML9.2*	*LOC_Os09g20350*	12,216,432	12,218,346	Ethylene-responsive transcription factor

### Marker Development and Validation

**FIGURE 8:**
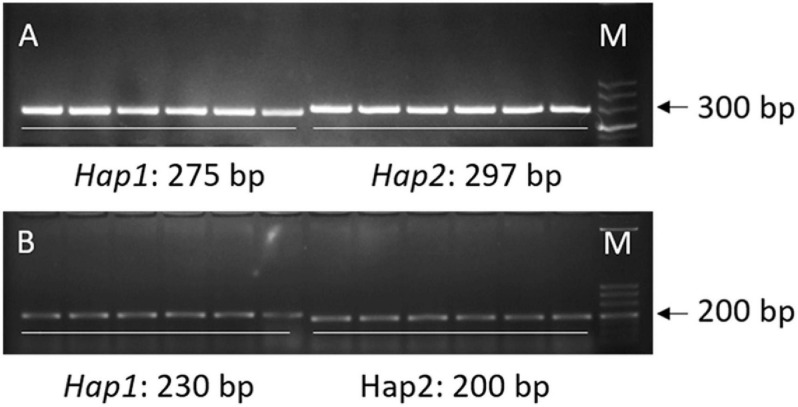
Two PCR gel-based markers based on the 22-bp InDel at chromosome 1 and the 30-bp InDel at chromosome 7. **(A)** Marker *Indel-Chr1:18932318*. **(B)** Marker *Indel-Chr7:15404166*.

For the loci (*qML1.3* and *qML7.2*) identified by both the QTL-seq and GWAS, a 22-bp insertion in the 18,932,318 bp for *qML1.3* at chromosome 1 and a 30-bp insertion in the 15,404,166 bp for *qML7.2* at chromosome 7 were selected for marker development to validate their effects. DNA fragments larger than 10 bp can be easily identified by agarose gel electrophoresis. To facilitate breeders utilizing the polymorphic information, we attempted to transfer all the InDels into PCR gel-based markers. The two molecular markers for *qML1.3* and *qML7.2* were named *Indel-Chr1:18932318* and *Indel-Chr7:15404166*, respectively (Table 5, Supplementary Table 9, and Figure 8).

**TABLE 5:** Primer information on the two developed markers for *qML1.3* and *qML7.2.*

Primer names	Primer sequence (5′–3′)	Amplicon size (bp)
*InDel-Chr1:18932318-F*	AGAACCCTTTTATCCTCATTA	275 (297)
*InDel-Chr1:18932318-R*	ACAAAGGGACTTGATGATGG	
*InDel-Chr7:15404166-F*	CACTAGCAAGAGTGCTCCCA	230 (200)
*InDel-Chr7: 15404166-R*	TTCTCAATACCCATGCCAAC	

*The amplicon size of ‘IR 145’ with the two markers were 275 bp and 230 bp respectively; whereas that of the female parent of Pop6, Kalasu, were 297 bp and 200 bp, respectively.*

**FIGURE 9:**
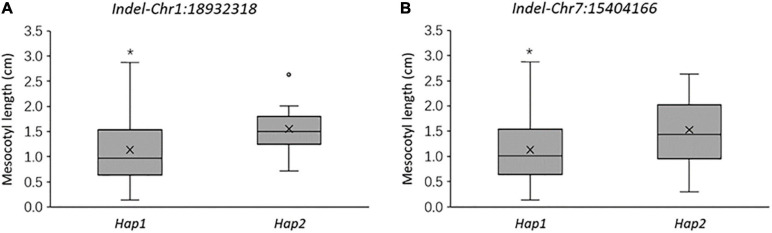
Mesocotyl length comparison of two haplotypes at 18,932,318 bp on chromosome 1 **(A)** and at 15,404,166 bp on chromosome 7 **(B)** in 140 accessions from the XI-1B population. *P < 0.05 (significant differences between the alleles).

X1-1B, with no obvious population structure (Supplementary Figure 4), was used to validate the effectiveness of the two markers. *Indel-Chr1:18932318* could divide the 140 accessions into two groups: the amplified fragment of group 1 was 275 bp, whereas that of group 2 was 297 bp (Figure 8A). Similarly, *Indel-Chr7:15404166* could divide the 140 accessions into two groups, with the amplified fragments being 230 and 200 bp (Figure 8B). An association analysis between the genotypes and phenotypes was conducted with 140 accessions from XI-1B of the 3K Resequencing Project, and significant differences in ML were detected. For *Indel-Chr1:18932318*, the mean ML of *Hap2* was significantly higher than that of *Hap1* by 0.42 cm (*P* < 0.05). For *Indel-Chr7:15404166*, the mean ML of *Hap2* was significantly higher than that of *Hap1* by 0.41 cm (*P* < 0.05) (Figure 9). ANOVA indicated that *Indel-Chr1:18932318*, *Indel-Chr7:15404166*, and the interaction between the two markers could explain ML variations at 2.68, 2.71, and 1.71%, respectively (Table 6).

**TABLE 6:** ANOVA for mesocotyl length in the XI-1B panel by two markers.

Marker	*df*	Sum of squares	Mean square	*F*-value	Pr(>*F*)
*Indel-Chr1:18932318*	1	1.178	1.776	2.984	0.0464
*Indel-Chr7: 15404166*	1	1.185	1.853	3.003	0.0354
(*Indel-Chr1:18932318*) × (*Indel-Chr7:15404166*)	2	0.737	0.737	1.867	0.174
Residuals	136	40.678	0.395		

## Discussion

Considerable variations were present in the two panels used for GWAS and the panel for validating the developed markers. The existence of a wide range of genetic variation has also been reported previously by many studies (Wu et al., 2005, 2015; Liu et al., 2020). Thus, identifying new loci and the associated markers is urgent and important for ML improvement in released cultivars, and breeding for long mesocotyl is feasible and has great potential. Although mesocotyl is a crucial rice developmental trait and is imminently required to improve crop adaptability to modern cultivation modes, QTL and genes are still not adequate, and more QTL and long mesocotyl donors need to be identified.

Traditional QTL mapping includes the construction of a mapping population and the phenotypic identification of a large number of individuals in the segregation population, which largely restricted the progress of QTL mapping (Salvi and Tuberosa, 2005). With the reduction of sequencing cost, QTL-seq has become an optimized strategy to rapidly identify the region harboring the genes/QTL of interest (Takagi et al., 2015). Besides, GWAS also provides a more representative gene pool and is an efficient tool that bypasses the time and expand to the developing population (Zhu et al., 2008). In total, 20 QTL for ML were identified in this study and indicated that the combination of GWAS and QTL-seq is an effective and reliable strategy for the rapid identification of major QTL for mesocotyl elongation.

In this study, 12 long mesocotyl accessions were selected as male parents to cross with the same female parent “IR 145,” a typical variety with a short mesocotyl. Six of the 14 unique loci identified by QTL-seq were detected in two or more populations, namely, *qML1.3*, *qML1.4*, *qML3.2*, *qML7.1*, *qML7.2*, and *qML9.1*, whereas the other loci were identified in one population. Also, the parents of Pop4, Pop5, Pop6, Pop7, Pop8, Pop9, and Pop12 were originated from the AUS panel, whereas the parents of Pop1, Pop2, Pop3, Pop10, and Pop11 were originated from the XI-1A panel. The loci identified in GWAS of the AUS and XI-1A panel were also detected in QTL-seq. These stable loci could be utilized in the breeding of long mesocotyl rice. Thus, QTL-seq with multiple populations based on the same parent could reduce the sequencing cost effectively while preserving a higher genetic diversity to identify the shared or unique loci and, thus, is a cost-saving and effective approach for genetic analysis of complex traits.

Six of the 14 QTL identified in this study overlapped with those identified previously by linkage mapping or GWAS. The QTL *qML7.2* has been reported at least five times using different biparental mapping populations or GWAS using different panels (Ouyang et al., 2005; Zhao et al., 2018). The QTL on chromosome 1 (*qML1.2*, 14.21–17.32 Mb), identified in Pop4 and the XI-1A panel, coincided with the loci detected by Lu et al. (2016) (marker: *seq-rs303*). *qML1.3* (18.59–21.12 Mb) and *qML1.4* (36.59–39.67 Mb) overlapped with the loci discovered by Wu et al. (2015) and Xiong et al. (2017). The *qML3.2* locus (27.21–32.30 Mb) on chromosome 3 crossed with the *qML3-2* isolated by GWAS based on 621 cultivars originated from the 3K Rice Resequencing Project (Zhao et al., 2018). QTL on chromosomes 8 (8.98–9.38 Mb), identified in the XI-1A panel, located in the same region with the loci identified from the Zhaxima/Hanhui 3 population (Yuldashev et al., 2012). Rohilla et al. (2020) have identified 20 genes significantly associated with anaerobic germination in a diverse panel with 94 deep-water rice genotypes of Assam. Of these, two most relevant genes, *OsXDH1* (*LOC_Os03g31550*) and *SSXT* (*LOC_Os12g31350*), have been identified that explain the higher phenotypic variability (*R*^2^ > 20%). The locus on chromosome 12 identified by Rohilla et al. (2020) overlapped with the *qML-AUS-12.1* identified in the AUS panel.

According to the results of GWAS and QTL-seq, eight genes involved in the biological metabolism of plant hormones, cell elongation, and cell division were selected as high-confidence candidate genes. A candidate gene (*LOC_Os07g13634*) on chromosome 7 encoding cytokinin-*N*-glucosyltransferase 1 was identified. CTKs are a class of plant hormones firstly identified as cell division-promoting factors and were subsequently identified as factors that control various processes in plant growth and development, including mesocotyl elongation (Cao et al., 2005). Hu et al. (2014) reported that there was an antagonistic relationship between SLs and CTKs in regulating mesocotyl elongation. CTKs downregulate the expression of *OsTCP5* and promote cell division, whereas SLs upregulate *OsTCP5* expression and inhibit mesocotyl elongation. Besides, CTKs play an important role in the biosynthesis of BRs, a group of steroid plant hormones essential to plant growth and development (Yuldashev et al., 2012). A candidate gene (*LOC_Os01g13200*) on chromosome 1 related to abscisic acid insensitive 8 was identified. The structure–activity of ABA analogs had a close relationship with the growth of rice mesocotyl and seedlings (Watanabe and Takahashi, 1999). ABA compounds regulate activity of the meristem localized near the coleoptile node (Watanabe et al., 2001). In addition, ABA interacts with GA in regulating rice mesocotyl elongation (Wu et al., 2002). A candidate gene (*LOC_Os06g09660*) on chromosome 6, which is an auxin response factor, was identified. Auxin is a simple small molecule based on the indole ring, which plays an important role in plant growth and development, such as cell differentiation, cell division, and cell elongation (Xu and Xue, 2012). Feng et al. (2017) reported that the exogenous IAA could promote mesocotyl elongation of etiolated rice seedlings for 2 days after germination in darkness.

Two candidate genes (*LOC_Os01g66100* and *LOC_Os04g33360*) on chromosomes 1 and 4 were related with the biosynthesis of GA, which encodes gibberellin 20 oxidase 2 and gibberellin 2-beta-dioxygenase 7, respectively. Liang et al. (2016) reported that GAs regulate mesocotyl cell elongation in rice. The destabilization of cortical microtubules (CMTs) increased the GA level and induced mesocotyl cell elongation, while polymerization of CMT showed the opposite result. The expressions of *GA20ox2*, *GA3ox2*, and *GID1* in GA biosynthesis and transferring pathway were regulated by CMTs (Liang et al., 2016). Two candidate genes (*LOC_Os07g28060* and *LOC_Os09g20350*) on chromosomes 7 and 9 were related with ethylene biosynthesis, which are an ethylene receptor and ethylene-responsive transcription factor, respectively. All of the above plant hormones have significant influence on mesocotyl elongation: SLs, CTKs, ABA, BRs, IAAs, JAs, and ecdysis-triggering hormone (ETH). The above hormones, except for ETH, have direct influence on mesocotyl elongation by affecting either cell division or elongation. Only ETH works as a signal to regulate cell elongation through the JA biosynthesis pathway. The expression of *GY1* was inhibited by ETH signaling in a MHZ7/OsEIN2-dependent way. When OsEIL2 binds to the *GY1* promoter, its activity was repressed directly, which further inhibited JA biosynthesis and promoted cell elongation (Xiong et al., 2017). A candidate gene (*LOC_Os03g56060*) on chromosome 3, a member of the CSLC9-cellulose synthase-like family C, was identified. Cellulose synthase is involved in plant growth and development, including the development of roots and stems and the elongation of root hair (Li et al., 2019; Moon et al., 2019). The expressions of eight candidate genes in different accessions indicated that these genes were all functional in regulating mesocotyl elongation.

For loci identified by GWAS and QTL-seq, based on the variations in *qML1.3* and *qML7.2*, functional markers *Indel-Chr1:18932318* and *Indel-Chr7:15404166* were developed to genotype the XI-1B panel. It is co-dominant, breeder-friendly, and can be easily implemented in the laboratory. Its potential value for the selection of long mesocotyl germplasm was validated by association analysis. *Indel-Chr1:18932318* and *Indel-Chr7:15404166*, identified with the marker, were associated with ML. Although frequencies of favorable alleles of the two loci were higher among XI-1A globally, further increases are still feasible in regions where the alleles occur at higher frequencies. Functional markers related to higher MLs so far reported are available for *OsGSK2* and *OsSMAX1* (Sun et al., 2018; Zheng et al., 2020). Marker-assisted selection (MAS) for longer mesocotyl breeding based on combinations of these favorable alleles will be more effective than selection for single markers. Our study provides two valuable functional markers for longer mesocotyl breeding. Our follow-up studies will focus on validating the effects of these QTL, decoding the mechanism on mesocotyl elongation, and utilizing the markers to assist breeding practice.

## Conclusion

Identifying QTL associated with mesocotyl elongation could accelerate genetic improvements of ML in rice breeding. QTL-seq of 12 F_2_ populations and GWAS of two diverse panels were conducted to identify QTL for ML. In total, 14 QTL were identified by QTL-seq and five loci explaining 5.3–14.6% of the phenotypic variations were identified by GWAS. Among these, six were stable across two or more populations and 10 were potentially novel loci. Besides, eight high-confidence candidate genes were identified involved in the biological metabolism of plant hormones, cell elongation, and cell division. In addition, two PCR gel-based markers (*Indel-Chr1:18932318* for *qML1.3* and *Indel-Chr7:15404166* for *qML7.2*) were validated and could be used as breeder-friendly markers in further breeding. This study provides new insights into the genetic regulation mechanism of rice mesocotyl elongation and could promote the rice breeding process.

## Data Availability Statement

The datasets to the Genome Sequence Archive can be found at https://ngdc.cncb.ac.cn/search/?dbId=&q=PRJCA005531, accession number PRJCA005531.

## Author Contributions

GY conceived and designed the experiments. YW, YM, HL, and CL performed the experiments. JL and YW analyzed the data. YW, JL, and GY wrote and revised the manuscript. All authors read and approved the final manuscript.

## Conflict of Interest

The authors declare that the research was conducted in the absence of any commercial or financial relationships that could be construed as a potential conflict of interest.

## Footnotes

1 www.fao.org

2 https://imagej.en.softonic.com/

3 https://www.berrygenomics.com/

4 http://rice.plantbiology.msu.edu/

5 http://broadinstitute.github.io/picard/

6 https://gatk.broadinstitute.org/hc/en-us

7 http://www.zzlab.net/GAPIT/

8 http://rice.plantbiology.msu.edu/cgi-bin/gbrowse/rice/

9 http://www.premierbiosoft.com/

10 http://www-01.ibm.com/software/analytics/spss/

## Supplementary Material

Supplementary Figure 1 Distribution of mesocotyl length in 12 F_2_ populations.

Supplementary Figure 2 Distribution of mesocotyl length in XI-1A, AUS and XI-1B panel.

Supplementary Figure 3 LD decay along the whole genome of the AUS and XI-1A panel.

Supplementary Figure 4 Population and kinship analysis for the XI-1B panel. **(A)** PCA analysis; **(B)** Kinship analysis.

Supplementary Table 1 Mesocotyl length of the accessions from XI-1A panel.

Supplementary Table 2 Mesocotyl length of the accessions from AUS panel.

Supplementary Table 3 Details of the next-generation sequencing data for 12 F_2_ populations.

Supplementary Table 4 Number of SNPs for the 12 F_2_ populations.

Supplementary Table 5 GWAS for mesocotyl length in the XI-1A panel.

Supplementary Table 6 GWAS for mesocotyl length in the AUS panel.

Supplementary Table 7 Candidate genes in the loci identified by QTL-seq and GWAS.

Supplementary Tables 8 Primer information for qRT-PCR of the candidate genes.

Supplementary Table 9 Mesocotyl length and allele information for developed markers in XI-1B panel.
