# Supplementary material for: Rapid Identification of QTL for Mesocotyl Length in Rice Through Combining QTL-seq and Genome-Wide Association Analysis
Source: Front Genet. 2021 Jul 19;12:713446. doi: 10.3389/fgene.2021.713446 (PMC8326918; doi:10.3389/fgene.2021.713446)
Supplement: Supplementary Figure 1 — Distribution of mesocotyl length in 12 F2 populations. [file Data_Sheet_1.docx]

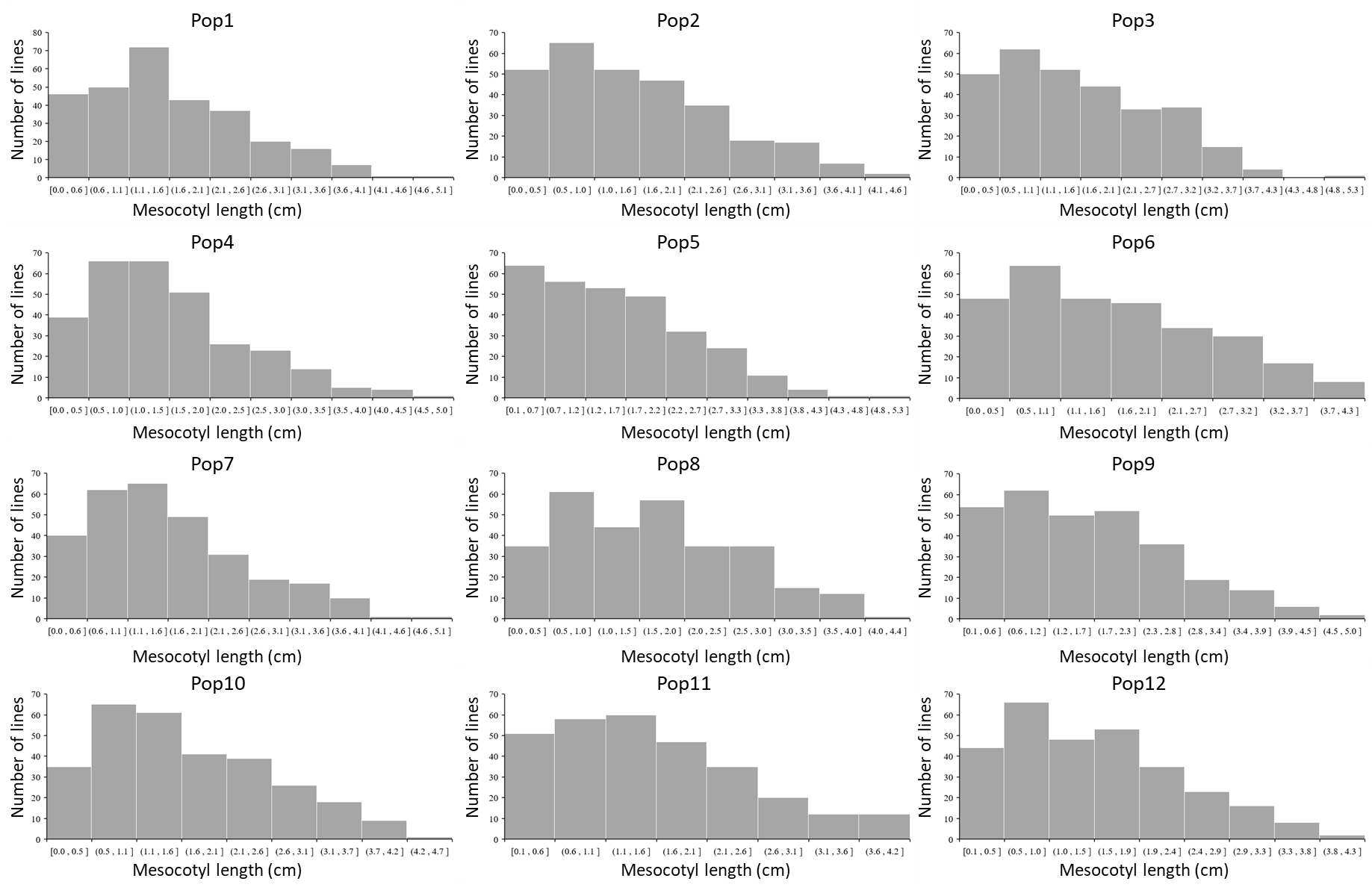


**Figure S1** Distribution of mesocotyl length in 12 F_2_ populations

**
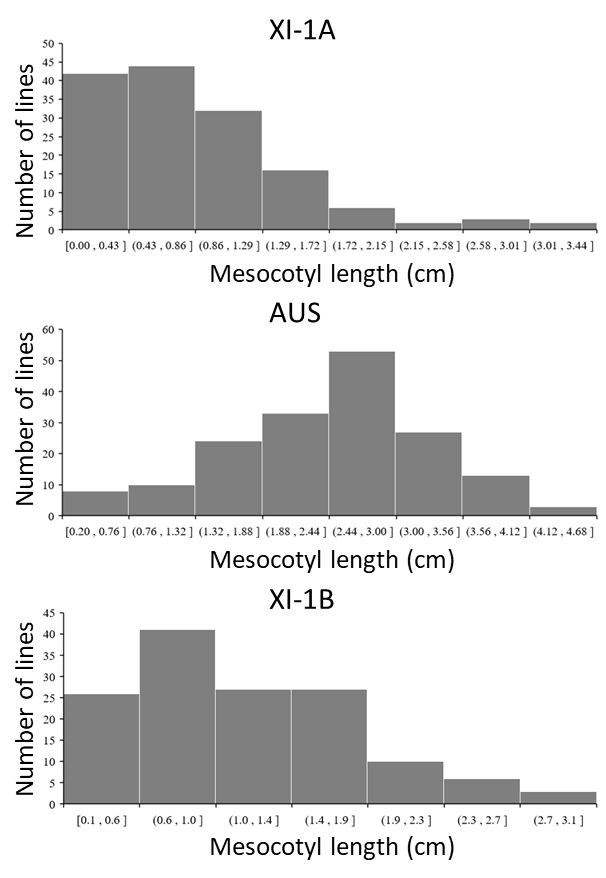
**

**Figure S2** Distribution of mesocotyl length in XI-1A, AUS and XI-1B panel

**
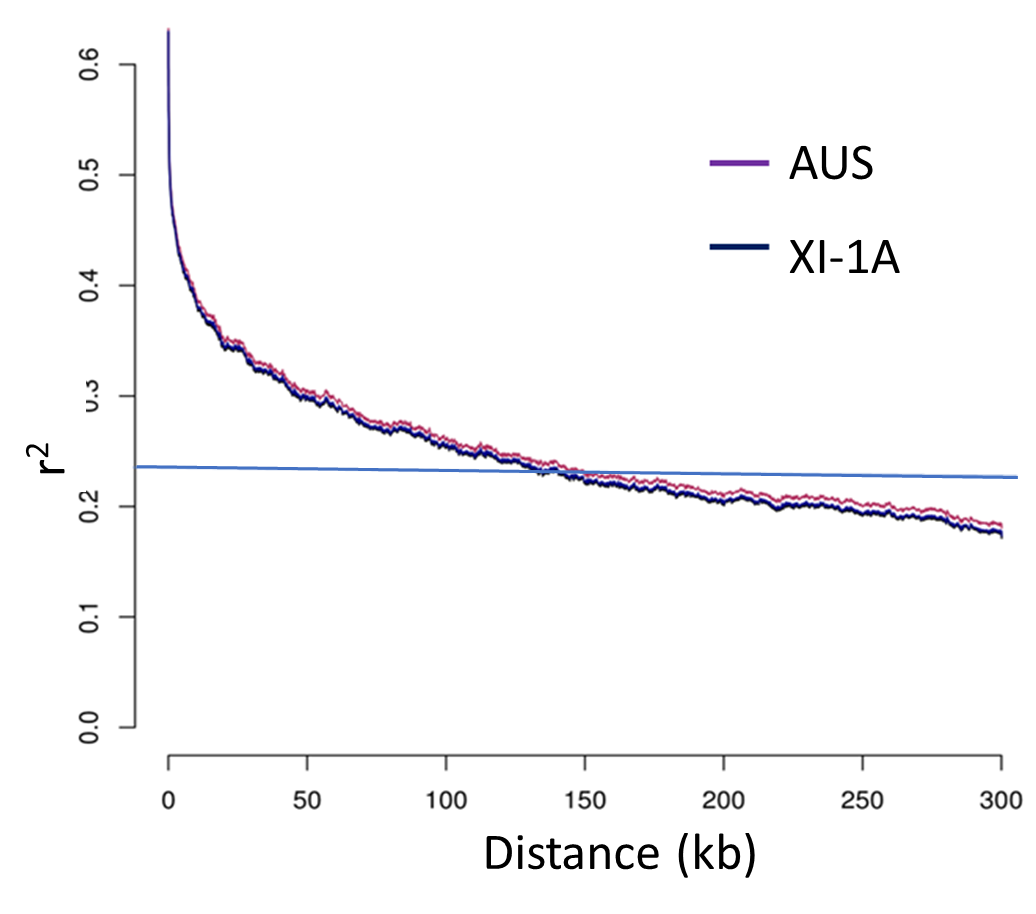
**

**Figure S3** LD decay along the whole genome of the AUS and XI-1A panel

**
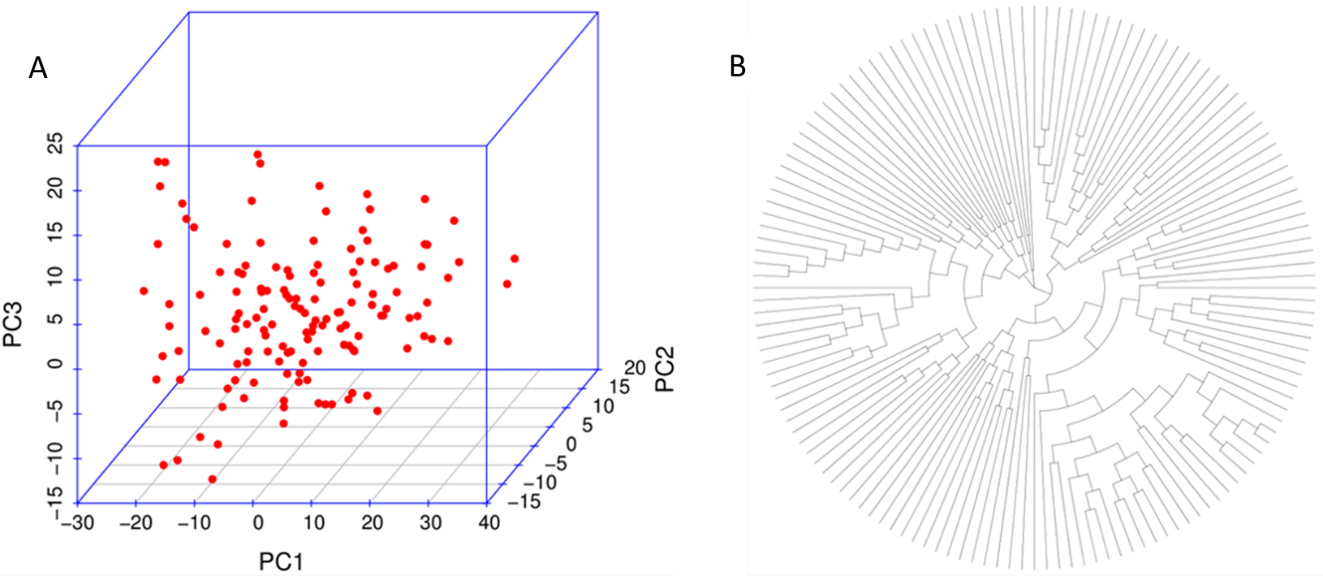
Figure S4** Population and kinship analysis for the XI-1B panel

A, PCA analysis; B, Kinship analysis
